# Feasibility and Preliminary Effects of a Culturally Adapted WHO IPV Training for Thai Healthcare Providers: A Quasiexperimental Pilot Study

**DOI:** 10.1155/nrp/6681793

**Published:** 2025-12-29

**Authors:** Tipparat Udmuangpia, Tina Bloom, Benjaporn Thitiyanviroj, Aimon Butudom, Wannaporn Kampila, Chiraporn Worawong, Supawadee Thaewpia

**Affiliations:** ^1^ Boromarajonani College of Nursing, Faculty of Nursing, Praboromarajchanok Institute, Khon Kaen, Thailand, bcn.ac.th; ^2^ Notre Dame of Maryland University School of Nursing, Baltimore, Maryland, USA; ^3^ Faculty of Nursing, Praboromarajchanok Institute, Nonthaburi, Thailand

**Keywords:** IPV screening, longitudinal research, pilot test, training program, WHO curriculum

## Abstract

**Aims:**

Intimate partner violence (IPV) is a significant yet underreported public health issue in Thailand. This study culturally adapted and piloted the World Health Organization (WHO) IPV training curriculum for Thai healthcare providers, evaluating its feasibility and preliminary effects on attitudes, subjective norms, perceived behavioral control, and screening behavior, guided by the theory of planned behavior (TPB).

**Design:**

Quasiexperimental, single‐group pilot study.

**Methods:**

A structured adaptation process (ADDIE model) localized the WHO curriculum to Thai cultural and healthcare contexts. Twenty‐two providers completed a 5‐day blended program (12‐h online theory, 18‐h practice with mentored sessions). Outcomes were measured at baseline, immediately post‐training, and 1‐month follow‐up using validated TPB‐based instruments. Analyses employed Wilcoxon signed‐rank tests, McNemar’s tests, and logistic regression.

**Results:**

Attitudes improved significantly (mean difference = 0.34; *p* = 0.005), and IPV screening behavior increased from 22.7% pretraining to 72.7% at 1 month (*p* < 0.001), with no decline between post‐training and follow‐up. Changes in subjective norms (*p* = 0.070) and perceived behavioral control (*p* = 0.416) were not statistically significant. Prior screening experience predicted screening at follow‐up (OR = 8.08; 95% CI: 1.53–42.78). Participants identified persistent barriers, including workload, time constraints, and family presence during consultations.

**Conclusion:**

The culturally adapted WHO IPV curriculum was feasible and acceptable and showed promising effects on attitudes and screening behavior among Thai providers. However, sustaining changes in subjective norms and perceived control requires post‐training organizational reinforcement.

**Summary**

• Implications for the profession and patient care:
◦ Our findings also supported the need for (1) effective training to improve the Thai healthcare response to intimate partner violence (IPV) and (2) maintaining practice changes through clear policy guidelines and organizational support. Future research should employ larger, randomized samples and mixed‐methods evaluation to further support scale‐up and implementation.
• Impact
◦ This study begins to address the need for effective training to improve the Thai healthcare response to IPV.
◦ We found preliminary evidence that our training can impact providers’ attitudes and screening behaviors.
◦ This study provides a foundation for future research and interventions to support Thai healthcare providers and ultimately, to help improve survivors’ health and safety.
• Reporting method
◦ We have adhered to the Strengthening the Reporting of Observational Studies in Epidemiology (STROBE) reporting checklist.
• Patient or public contribution
◦ This study did not include patient or public involvement in its design, conduct, or reporting.

## 1. Introduction

IPV remains a pervasive global health challenge with profound implications for women’s physical, mental, and social wellbeing. According to the World Health Organization [1], approximately 30% of women worldwide have experienced physical and/or sexual violence perpetrated by intimate partners. In Thailand, IPV prevalence varies significantly by region and demographic characteristics. Chuemchit et al. [2] reported 15% of married or cohabiting women aged 20–59 years had experienced psychological, physical, and/or sexual violence in their lifetime. However, Garcia‐Moreno et al. [3] identified substantially higher rates in Bangkok, with 36.1% of women reporting experiences of physical and/or sexual partner violence.

The pattern of IPV is particularly concerning, as it typically manifests in recurring episodes rather than isolated incidents. Women who experience one form of domestic violence frequently endure multiple forms of repeated abuse [4]. This cyclical pattern significantly affects victims’ health outcomes, with four out of five affected women reporting adverse impacts on their physical and mental health, as well as their employment status [4]. IPV is especially critical among vulnerable populations such as pregnant women in Thailand. A study by Saito et al. [5] documented that pregnant women reported experiencing abuse, with psychological abuse being the most prevalent (53.7%), followed by physical (26.6%) and sexual abuse (19.2%).

The health consequences of IPV extend beyond immediate physical injuries. Victims often develop chronic health conditions, including persistent pain, gastrointestinal disorders, and cardiovascular problems [6]. Mental health effects are equally severe, with IPV survivors showing significantly higher rates of depression, anxiety, post‐traumatic stress disorder, and suicidal ideation [7, 8].

Given these severe health impacts, effective IPV screening is a critical public health intervention. The WHO recommends universal screening for domestic violence among women accessing healthcare services, particularly during antenatal care, family planning consultations, postnatal visits, gynecological examinations, and emergency department presentations [9]. Systematic screening serves multiple purposes: It identifies victims who might not otherwise disclose abuse, raises awareness of IPV as a health issue, and facilitates timely interventions that mitigate immediate and long‐term consequences [10–13].

For pregnant women specifically, IPV screening carries additional significance due to its potential impact on both maternal and fetal outcomes. Effective screening and subsequent interventions can help monitor fetal wellbeing, reduce prenatal depression, and prevent adverse birth outcomes such as preterm delivery and low birthweight [8, 14, 15]. However, implementing screening programs requires careful consideration of contextual factors to avoid unintended negative consequences, such as increased anxiety, stigmatization, or psychological distress, particularly when screening methods are inappropriate or healthcare providers lack adequate training [16–19].

In Thailand, despite many women’s health and antenatal services endorsing routine screening, implementation remains uneven, especially for pregnant women, leaving a clear, actionable gap for system‐wide adoption and fidelity. Limited data availability on IPV screening rates and the absence of routine recording mechanisms contribute to perceptions of low screening rates. Several systemic barriers further impede effective implementation. Healthcare providers frequently report insufficient knowledge, understanding, and training regarding IPV screening and management [20, 21].

This deficiency is compounded by ambiguity regarding professional roles and responsibilities, with nurses, physicians, and social workers often expressing uncertainty about their obligations in IPV screening processes [19, 22]. Furthermore, the lack of standardized screening tools and clear referral pathways presents additional barriers to consistent practice [23–25].

A fundamental challenge lies in inadequate coverage of IPV content within medical and nursing education curricula. Alshammari et al. [26] highlight this significant educational gap, noting that many healthcare professionals enter clinical practice with minimal formal training on recognizing and responding effectively to IPV. This educational deficiency underscores the critical need for specialized training programs that equip healthcare providers with necessary competencies.

To address these challenges, the WHO developed the Listen, Inquire, Validate, Enhance safety, and Support (LIVES) model, a comprehensive training framework for frontline healthcare workers [27]. This evidence‐based approach encompasses diverse educational methodologies, including lectures, interactive workshops, case studies, and practical skill‐building exercises designed to enhance providers’ knowledge, attitudes, and confidence in assisting IPV survivors.

While the WHO curriculum provides a valuable foundation, effective implementation requires adaptation to specific cultural, social, and healthcare system contexts. Thailand’s unique cultural norms regarding family dynamics, gender roles, and help‐seeking behaviors necessitate a tailored approach that resonates with local healthcare providers and the communities they serve. Although IPV is recognized as a critical health issue, limited evidence exists on the effective implementation of IPV screening programs in Thailand, particularly within antenatal and women’s healthcare settings. Therefore, we aimed to evaluate the feasibility of a culturally adapted WHO IPV curriculum among Thai healthcare providers, addressing the critical need for contextually tailored interventions and contributing to bridging existing implementation gaps.

**Figure 1:**
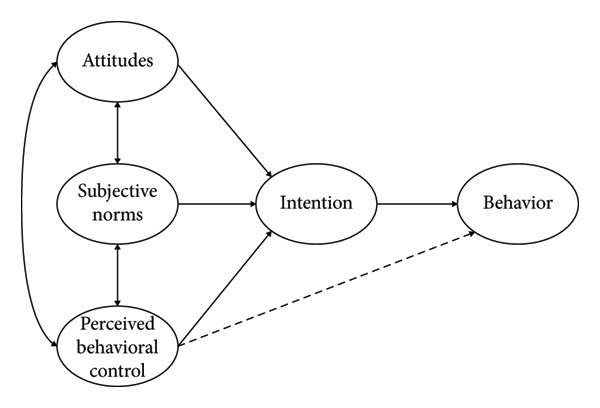
The component of theory of planned behavior (TPB) [28].

The theory of planned behavior (TPB) [28, 29] provides a robust framework for understanding and influencing clinical behavior change. By targeting attitudes, subjective norms, and perceived behavioral control, training can increase providers’ intention and ability to conduct IPV screening. The hypothesized relationships among TPB constructs are presented in Figure 1. However, a few studies in Thailand have evaluated IPV training interventions explicitly grounded in TPB.

The study was guided by the TPB, proposed by Ajzen [28, 29], a robust framework for understanding and influencing behavioral change among healthcare professionals. TPB was specifically chosen for its demonstrated efficacy in predicting and altering healthcare professionals’ clinical behaviors, providing a suitable framework to analyze attitudinal and practical changes resulting from training interventions. According to TPB, behavior is determined by three key components: attitudes, subjective norms, and perceived behavioral control. Within this study’s curriculum development context, these components manifested as follows:

*Attitudes*: Fostering positive attitudes among healthcare professionals toward IPV screening and response by emphasizing its critical importance for patient health and their professional responsibility in addressing the issue.

*Subjective norms*: Shaping participants’ perceptions of professional expectations and creating a supportive peer environment to establish IPV screening as a standard of care, aligning with institutional priorities.

*Perceived behavioral control:* Enhancing participants’ self‐efficacy and confidence through comprehensive knowledge transfer and practical skill development, enabling effective IPV screening and survivor support.

This theoretical framework guided both the adaptation of the WHO curriculum to the Thai context and the evaluation of its effectiveness in changing healthcare providers’ screening practices.

## 2. Methods

### 2.1. Study Design

This study employed a quasiexperimental, longitudinal, pretest–posttest, single‐group pilot design to evaluate the feasibility, acceptability, and potential effectiveness of a training program aimed at improving IPV screening by healthcare providers in Thailand. The intervention consisted of delivering a structured training program adapted from the WHO curriculum, with assessments conducted at three distinct time points: pretraining (baseline), immediately post‐training, and 1 month post‐training. This longitudinal approach allowed for evaluating both immediate and sustained effects, which are critical for determining the program’s practicality and potential for integration into the Thai healthcare system.

### 2.2. Participants and Recruitment

We recruited participants via convenience sampling through targeted social media outreach (e.g., hospital Facebook pages, Line groups) and official correspondence with local healthcare facilities. Eligible participants included nurses, social workers, public health officers, and other healthcare providers aged 22–60 years, of any gender, who were actively working in departments related to women’s care and domestic violence services (e.g., antenatal care, gynecology, postpartum, outpatient, emergency, and One‐Stop Crisis Centers). Additional inclusion criteria required at least 1 year of professional experience, proficiency in the Thai language, and willingness to complete the full training program and all follow‐up assessments. Recruitment occurred in August 2022. From an initial pool of 26 healthcare providers who expressed interest, 22 met the eligibility criteria and enrolled in the study.

### 2.3. Intervention

#### 2.3.1. Curriculum Development and Adaptation

We adapted *Caring for Women Subjected to Violence: A WHO Curriculum for Training Health-Care Providers* (2019) using ADDIE [30]: (1) Analysis: rapid needs assessment (policy review, service mapping, provider‐reported barriers); (2) Design: co‐design with clinicians to localize language, scenarios, and referral pathways; (3) Development: Thai translation/back‐translation, scripts reflecting Thai communication norms, privacy‐first workflow, and a local resource directory; (4) Implementation: blended delivery (12‐h theory, 18‐h practice); (5) Evaluation: expert validation and pilot feedback to refine job aids and scripts.

The development process included sequential phases, beginning with a situational analysis to assess specific needs and contextual factors relevant to IPV screening in Thailand. This was followed by collaborative design sessions and extensive literature reviews conducted by a multidisciplinary research team with expertise in IPV and healthcare delivery. Content development involved translating materials into Thai in collaboration with English‐speaking professionals and IPV researchers, drafting contents, and conducting multiple revisions based on team consensus (see Table 1). The draft curriculum then underwent expert validation by a panel of specialists in IPV and women’s health services, assessing content accuracy, appropriateness of screening tools, and cultural suitability. Expert validation: A five‐expert panel (IPV nurse specialist, obstetrician, emergency nurse leader, social worker, implementation scientist) used a 4‐point relevance scale to compute I‐CVI and S‐CVI/Ave. Items below the ≥ 0.78 I‐CVI threshold were revised and re‐reviewed. Finally, we pilot‐tested the curriculum with 22 healthcare providers to provide a preliminary evaluation of effectiveness, acceptability, and feasibility, with iterative refinements made based on participant feedback and evaluation by the research team.

**Table 1:** Comparative between the WHO curriculum and the Thai cultural adaptation.

WHO curriculum element	Thai cultural adaptation	Rationale in Thai context
Overall duration and delivery	Extended from 3 ⟶ 5 days; blended format (12‐h online theory; 18‐h practice including 2 onsite practice days at workplaces) + 2‐h mentor meeting per group	IPV screening is new and sensitive in Thailand; hands‐on practice in real settings builds confidence and safety
LIVES first‐line support	Thai phrasing emphasizing empathy/nonblaming; explicit privacy guidance when family accompanies patient	Align with family presence, and stigma concerns; increase disclosure safety
Cultural introduction to screening	Thai‐language video intros that normalize asking about IPV (e.g., “during pregnancy we ask everyone…”) before questions	Reduce shame/stigma; provide culturally acceptable opening
Skills practice format	Video‐based role‐plays + feedback (instead of mixed online/onsite simulations) with structured debriefs	Online delivery constraints; maintain reflective practice and standardization
Screening workflow and privacy	Privacy‐first protocol (ensure 3–5 min alone); use Universal Education handout when privacy not possible; brief safety check	Frequent family accompaniment; time pressure; safety
Referral pathways	Hospital social work, One‐Stop Crisis services, and local NGOs mapped; one‐page QR directory created	Clarity and speed for referrals in short visits
Substance‐use content	Added module on substance use (including during pregnancy) with brief counseling and referral guidance	High local prevalence; strong intersection with IPV
Integrated one‐page tool	Single‐page screening tool covering IPV, mental health, and substance use; used in training and practice	Streamlines workflow; lowers time burden; improves adoption
Translation and readability	Thai translation with back‐translation and plain‐language adjustments	Accuracy and clarity for multidisciplinary teams
Mentorship structure	2‐h small‐group mentoring with the research team during the practice phase	Sustain skills, problem‐solve real barriers, build self‐efficacy

#### 2.3.2. Training Program Structure

The 5‐day training program (30 h total) consisted of two complementary components delivered in a blended format (online theoretical sessions and in‐person practical training at participants’ clinical sites). We chose this blended approach to balance theoretical knowledge with practical skill acquisition, considering providers’ limited availability for extended off‐site training.

The first component included online theoretical training conducted over 2 days (12 h total). Adaptations to the original WHO curriculum included culturally relevant scenarios for case studies, role‐play activities reflecting typical interactions between Thai healthcare providers and female patients, and localization of referral pathways aligning with available community resources. Content emphasized women‐centered clinical care approaches, identification of IPV survivors, first‐line support using the WHO’s LIVES approach, compassionate and empathic provider–patient communication, reflective exercises examining attitudes toward survivors, and understanding survivors’ experiences from their perspectives.

The second component involved practical training conducted over 3 days (18 h total). This practical component included hands‐on practice with actual clients, application of IPV screening protocols, provision of essential clinical care to survivors (e.g., counseling), referral to local support resources, and follow‐up sessions addressing implementation challenges. The adapted curriculum emphasized integrating IPV screening with substance abuse screening where relevant, alongside appropriate counseling and referral practices. Special attention was given to promoting compassionate care and establishing linkages with local resources aligned with Thailand’s healthcare system structure.

### 2.4. Ethical Considerations

This study was approved by the Institutional Review Board of Boromarajonani College of Nursing, Khon Kaen, with approval number IRB‐BCNKK‐56‐2021 (December 20, 2021). Online survey completion was voluntary and fully informed. The participants were able to withdraw at any time during the study period.

### 2.5. Measures

*Sociodemographic variables*. A sociodemographic questionnaire collected participants’ age, gender, previous IPV‐related training, prior experience screening women for IPV, and personal experiences of IPV (self, family members, or friends).

*Exposures to IPV training, IPV screening, and IPV experience.* Participants’ exposure to IPV‐related practices and experiences was assessed through binary (yes/no) questions, including: “Have you ever observed healthcare professionals screening for IPV?” “Is an IPV screening tool available at your clinical site?” and “Was an IPV screening tool available in your department?” Additional items addressed participants’ own experiences screening for IPV and personal exposure to IPV, measured using the question: “Have you ever experienced physical, sexual, or emotional violence in an intimate partner relationship?” (yes/no), as well as exposure among family members (yes/no).

*The IPV Screening Intention (IPVSI)* instrument. The IPVSI instrument was adapted from the IPVSI Instrument for Nursing Students (IPVSI‐NS [31]). The adapted IPVSI underwent rigorous validation involving expert consultation and pretesting among a representative sample of healthcare providers to ensure cultural appropriateness, clarity, and relevance. This Thai‐language instrument assessed IPV screening intentions among healthcare providers, explicitly incorporating the TPB constructs. The IPVSI comprises 36 items divided into three subscales: attitudes (14 items) measuring participants’ evaluation of IPV screening importance and value; subjective norms (9 items) assessing perceived social pressure regarding IPV screening; and perceived behavioral control (13 items) gauging perceived ease or difficulty in performing IPV screening. All items used a 5‐point Likert scale (1 = strongly disagree, 5 = strongly agree), with eight items reverse‐scored. Higher scores indicated greater levels of each construct. The IPVSI’s content validity was confirmed by three IPV research specialists, and reliability testing conducted among 30 healthcare personnel from a local hospital in Khon Kaen Province yielded a Cronbach’s Alpha of 0.76, indicating acceptable internal consistency.

*Intention to Screen.* Intention to screen was defined as healthcare providers’ motivation to perform IPV screening, measured with a single binary question: “I intend to screen female patients for IPV” (“intention” vs. “no intention”).

*Screening behavior.* Screening behavior was defined as healthcare providers’ actual IPV screening performance, assessed using a single binary question: “Have you screened for IPV in female clients who use services in hospitals or health service centers to ensure those experiencing violence receive care?” (“have screened” vs. “have not screened”).

Data were collected consistently at three time points pretraining (baseline), immediately post‐training, and 1 month post‐training to ensure comparability and robust longitudinal evaluation.

### 2.6. Data Analysis

We analyzed data using SPSS Version 24.0 [32]. The independent variable was the IPV screening training program, while dependent variables included attitudes, subjective norms, perceived behavioral control, intention to screen, and actual screening behavior.

The analysis began with descriptive statistics. Demographic data were summarized using frequencies, percentages, means, standard deviations, medians, ranges, and percentiles. Bivariate relationships among demographic characteristics, TPB components (attitudes, subjective norms, perceived behavioral control), and intention to screen were examined using Spearman correlation coefficients, with statistical significance set at *p* < 0.05.

Logistic regression was employed to identify significant predictors of actual IPV screening behavior, emphasizing practical implementation factors and prior screening experiences, which may significantly influence behavioral adoption in clinical settings.

For longitudinal analysis, the Wilcoxon signed‐rank test was used to examine changes in continuous variables (attitudes, subjective norms, perceived behavioral control) across the three assessment points (pretraining, post‐training, and 1‐month follow‐up). This nonparametric approach was chosen due to the small sample size (*N* = 22), repeated measurement design, and non‐normal distribution of the data. Additionally, McNemar’s test evaluated changes in screening behavior (a dichotomous outcome) across the same time points. Nonparametric tests (Wilcoxon signed‐rank and McNemar’s tests) provided robust measures of within‐subject changes over time, ensuring accuracy despite sample size and data distribution limitations.

The research hypothesis proposed that participants completing the training program would demonstrate significantly improved scores in attitudes, subjective norms, perceived behavioral control, and screening behavior post‐training compared to baseline measures.

## 3. Results

### 3.1. Participant Characteristics

Participants (*N* = 22) had a retention rate of 100%, a mean age of 40 years (SD = 8.06), and were predominantly female (95.45%). The majority (59.09%) had never received any education, lectures, or training related to IPV in their workplace. Most participants (68.18%) indicated that IPV screening forms were not available for female clients in their workplace. Approximately two‐thirds (63.64%) reported no prior experience conducting IPV screening. Regarding personal experiences with IPV, 81.82% of participants reported they had never personally experienced physical, sexual, or psychological violence from a current or former partner, and 68.18% had never experienced such violence from family members or friends (see Table 2).

**Table 2:** Participant demographics.

Characteristics	Number (*N* = 22)	Percentage
Age (years)		
Mean (standard deviation)	40 (8.06)	
Gender		
Female	21	95.45
Male	1	4.55
Previous training or education on IPV in workplace		
Yes	9	40.91
No	13	59.09
Availability of IPV screening forms for female clients at workplace		
No	15	68.18
Yes	6	27.27
Don’t know	1	4.55
Previous experience screening for IPV		
Yes	8	36.36
No	14	63.64
Personal experience of physical, sexual, or psychological violence from a current or former partner		
Yes	4	18.18
No	18	81.82
Personal experience of physical, sexual, or psychological violence from family members or friends		
Yes	7	31.82
No	15	68.18

### 3.2. Changes in Attitudes, Subjective Norms, and Perceived Behavioral Control After One Month

One month after implementing the training, participants demonstrated significantly increased awareness of practical barriers affecting IPV screening effectiveness. Specifically, participants showed heightened recognition that the presence of family members during screening could interfere with the accuracy of information obtained (score increased from 4.18 to 4.59). Additionally, participants reported greater awareness that heavy nursing workloads (4.13–4.50), high patient volumes (4.27–4.59), and limited time available for each patient (4.27–4.50) were substantial barriers to effective screening practices.

Participants also expressed enhanced readiness and confidence in their screening abilities. They reported improved personal readiness to perform IPV screening (4.45–4.63), greater ease in conducting screening activities (4.54–4.63), and increased recognition of the critical importance of adequate nursing knowledge and skills for effective screening (4.72–4.77).

Moreover, participants demonstrated stronger awareness regarding the advocacy aspects of IPV screening. They increasingly viewed screening as a means of highlighting IPV as a critical public health issue (4.63–4.77) and strengthened their belief that information obtained from screening could support safety planning for survivors (4.77–4.81).

However, certain areas experienced slight declines in 1 month post‐training. Participants’ perceptions of workplace support showed reductions, including clarity of workplace policies and guidelines related to IPV screening (from 4.45 to 4.22), support received from supervisors (4.45–4.31), and support from nursing colleagues (4.45–4.36). Additionally, some attitudes toward screening experienced minor decreases, particularly perceptions of screening as an effective assessment process (4.72–4.59) and the importance of gentle and empathic care in encouraging survivor disclosures (4.68–4.59) (see Table 3).

**Table 3:** Attitudes, subjective norms, and perceived behavioral control: Post‐training and 1 month after training.

Attitudes, subjective norms, and perceived behavioral control	Pretraining		Post‐training		1 month after training	
	$\overset{¯}{X}$	SD	$\overset{¯}{X}$	SD	$\overset{¯}{X}$	SD
1. Screening for intimate partner violence among female clients is a good assessment process	4.50	0.59	4.72	0.45	4.59	0.90
2. Nurses play a crucial role in screening for intimate partner violence among female clients	4.63	0.49	4.77	0.42	4.81	0.39
3. Screening for intimate partner violence is an activity that you can perform easily and comfortably	4.27	0.70	4.54	0.73	4.63	0.58
4. Screening for intimate partner violence is beneficial, allowing you to identify risks and violence that may cause health problems for clients under your care	4.54	0.50	4.81	0.39	4.72	0.45
5. Screening for intimate partner violence provides an opportunity to help and arrange health services for those who have experienced or are in situations of family violence	4.63	0.49	4.81	0.50	4.77	0.42
6. Screening for intimate partner violence can provide information for planning assistance to ensure safety for those experiencing violence	4.63	0.49	4.77	0.42	4.81	0.39
7. Screening for intimate partner violence is an activity that can educate female clients about intimate partner violence	4.40	0.50	4.81	0.39	4.81	0.39
8. Screening for intimate partner violence is an activity that helps emphasize and push intimate partner violence issues into public and healthcare awareness	4.40	0.50	4.63	0.58	4.77	0.42
9. Theoretical and practical education about intimate partner violence in nursing curriculum plays an important role in promoting and preparing you for intimate partner violence screening	4.54	0.50	4.72	0.55	4.77	0.42
10. Female clients lack confidence and trust to disclose intimate partner violence to you	3.40	1.05	3.68	1.32	3.95	1.39
11. Screening for intimate partner violence may cause clients to feel embarrassed	3.04	1.21	3.72	1.42	3.72	1.35
12. Screening for intimate partner violence will cause clients significant anxiety if they are identified as victims of violence	3.27	1.16	3.77	1.15	3.86	1.39
13. Screening for intimate partner violence is a very time‐consuming activity	3.45	1.10	4.13	1.16	4.18	1.05
14. Screening for intimate partner violence may increase risk to clients if female clients receive insufficient and noncomprehensive assessment	3.40	1.22	4.00	1.19	4.09	1.10
15. Nurses in your training facility support and expect you to conduct intimate partner violence screening	4.09	0.75	4.40	0.79	4.40	0.73
16. Your supervisors or department heads support and expect you to conduct intimate partner violence screening	4.22	0.68	4.45	0.73	4.31	0.99
17. Multidisciplinary professionals (doctors, nurses, social workers, etc.) in your workplace support and expect you to conduct intimate partner violence screening	4.00	0.61	4.40	0.73	4.36	0.78
18. Fellow nurses who work with you support and expect you to conduct intimate partner violence screening	4.04	0.65	4.45	0.67	4.36	0.78
19. Opinions of fellow nurses at your workplace regarding intimate partner violence screening are important to your decision to screen	3.86	0.88	4.13	0.88	4.09	1.10
20. Opinions of multidisciplinary professionals in your workplace regarding intimate partner violence screening are important to your decision to screen	3.81	0.85	4.04	1.17	4.04	1.13
21. Opinions of department heads regarding intimate partner violence screening are important to your decision to screen	3.81	0.85	4.13	0.99	4.09	1.10
22. Opinions of fellow nurses regarding intimate partner violence screening are important to your decision to screen	3.77	0.86	4.18	1.00	4.13	1.08
23. Clear policies and guidelines in your workplace regarding intimate partner violence screening play an important role in your decision to screen	4.22	0.68	4.45	0.67	4.22	0.97
24. Gentle and friendly care from nurses will help female clients become more willing to disclose intimate partner violence	4.59	0.50	4.68	0.47	4.59	0.59
25. Trust relationships between nurses and female clients will make intimate partner violence screening go smoothly and yield accurate information	4.63	0.49	4.72	0.55	4.77	0.42
26. Regular training on intimate partner violence screening will help improve your screening effectiveness	4.68	0.56	4.63	0.65	4.72	0.55
27. You think your workplace has adequately prepared you with knowledge and skills for screening intimate partner violence among female clients	3.95	0.65	4.31	0.83	4.40	0.85
28. Private, safe, and secure locations will facilitate effective intimate partner violence screening and yield accurate information	4.31	0.71	4.63	0.49	4.63	0.65
29. Working as a team with both nurses and multidisciplinary professionals will make intimate partner violence screening more effective	4.50	0.67	4.68	0.47	4.72	0.63
30. Effective intimate partner violence screening requires screeners to have sufficient knowledge and skills	4.50	0.59	4.72	0.55	4.77	0.52
31. Adequate and diverse support resources for victims of violence will promote intimate partner violence screening	4.40	0.66	4.54	0.73	4.54	0.73
32. Having family members present during intimate partner violence screening may interfere and prevent obtaining accurate information	4.22	0.81	4.18	0.90	4.59	0.73
33. The high workload assigned to nurses may affect intimate partner violence screening	4.18	0.73	4.13	0.94	4.50	1.05
34. Having many clients in the clinic or ward may affect intimate partner violence screening	4.36	0.73	4.27	0.93	4.59	0.90
35. Limited time for each nursing activity is a barrier to intimate partner violence screening	4.36	0.65	4.27	0.93	4.50	0.91
36. You are ready to screen for intimate partner violence in female clients	4.31	0.71	4.45	0.59	4.63	0.58
Total	4.34	0.48	4.41	0.48	4.46	0.60

### 3.3. Changes in Theoretical Components of IPV Screening Behavior

Immediately following the training intervention, participants demonstrated meaningful changes in attitudes toward IPV screening. The mean score for attitudes significantly increased from 4.08 (SD = 0.49) pretraining to 4.42 (SD = 0.51) post‐training, yielding a statistically significant mean difference of 0.34 (*t* = 3.43, *p* = 0.005). However, changes in subjective norms were modest, with the mean score increasing from 4.09 (SD = 0.54) to 4.37 (SD = 0.62), which was not statistically significant at the 0.05 level (*t* = 1.90, *p* = 0.0701). Similarly, perceived behavioral control scores exhibited a slight, nonsignificant increase from 4.34 (SD = 0.48) to 4.44 (SD = 0.46; mean difference = 0.09; *t* = 0.83, *p* = 0.4159) (see Table 4).

**Table 4:** Changes in theoretical components of IPV screening behavior.

Comparison categories	Pretraining score		Post‐training score		1 month after training		$\overset{¯}{D}$ (mean difference)	*t*	*p*‐value
	$\overset{¯}{X}$	SD	$\overset{¯}{X}$	SD	$\overset{¯}{X}$	SD			
Attitudes	4.08	0.49	4.42	0.51	4.44	0.58	0.34	3.43	0.005
Subjective Norms	4.09	0.54	4.37	0.62	4.46	0.67	0.27	1.90	0.0701
Perceived behavioral control	4.34	0.48	4.44	0.46	4.46	0.56	0.09	0.83	0.4159

Regarding screening behavior, the results showed a remarkable improvement. Prior to training, only 22.7% of participants performed IPV screening. Immediately post‐training, this increased dramatically to 68.2% and further rose slightly to 72.7% at the 1‐month follow‐up.

McNemar’s test indicated a statistically significant improvement from pretraining to 1‐month follow‐up (*χ* ^2^ = 11.00, *p* < 0.001), with 22 participants adopting screening practices and none discontinuing them. Importantly, screening behaviors remained stable between post‐training and the 1‐month follow‐up (*χ* ^2^ = 0.33, *p* = 0.564), suggesting sustained behavioral change over time (see Table 5 and 6).

**Table 5:** Changes in screening behavior across training phases (*N* = 22).

Comparison	McNemar’s test chi‐square	*p*‐value	Change direction
Pretraining to one‐month follow‐up	*χ* ^2^ = 11.00	< 0.001	11 increased, 0 decreased
Post‐training to one‐month follow‐up	*χ* ^2^ = 0.33	0.564	2 increased, 1 decreased

**Table 6:** Frequency and percentage of intimate partner violence screening behavior across training phases (*N* = 22).

Period	Performed screening (*n*, %)	Did not perform screening (*n*, %)
Pretraining	5 (22.7%)	17 (77.3%)
Post‐training	15 (68.2%)	7 (31.8%)
One‐month follow‐up	16 (72.7%)	6 (27.3%)

### 3.4. Predictors of IPV Screening Behavior

Further analysis to identify predictors of screening behavior showed a significant association between prior IPV screening experience and current screening practice (*p* = 0.014). Specifically, healthcare providers with previous IPV screening experience were 8.1 times more likely to screen female clients for IPV compared to providers without prior experience. This finding underscores the influential role of prior familiarity and practical experience with screening procedures in driving the adoption of screening behaviors (see Table 7).

**Table 7:** Predicting intimate partner violence screening behavior.

Variable	Coefficient	SE	Wald	*p*‐value	Odds ratio	(95% CI)
Previous experience screening for IPV	2.09	0.85	2.46	0.014	8.08	0.42–3.76

## 4. Discussion

This quasiexperimental longitudinal pilot study provides novel preliminary evidence on the feasibility, acceptability, and preliminary effects of a culturally adapted training program, marking the first implementation of the “*Caring for Women Subjected to Violence: A WHO Curriculum*” [27] in Thailand. Using a pretest–posttest single‐group design with measurements at three time points (pretraining, post‐training, and 1‐month follow‐up), this research advances understanding of how structured interventions influence attitudes, subjective norms, perceived behavioral control, and screening behaviors over time within the context of a middle‐income country. The significant improvements in participants’ attitudes and screening behaviors, along with the identification of prior screening experience as a key predictor, provide novel insights for optimizing IPV training among Thai healthcare providers.

The training program, grounded in the WHO curriculum’s emphasis on women‐centered care and the LIVES approach, significantly enhanced participants’ attitudes toward IPV screening (mean difference = 0.34, *p* = 0.005). This finding aligns with recent evidence that targeted education can shift healthcare provider perceptions, emphasizing IPV screening as a recognized priority within healthcare [17, 19, 33, 34]. Uniquely, this study revealed increased participant awareness of practical screening barriers, such as family presence (4.18–4.59) and heavy workloads (4.13–4.50), reflecting the program’s effectiveness in combining skills training with contextual sensitivity. This dual impact distinguishes our findings from prior studies that typically focus solely on attitudinal shifts without addressing practical barriers in resource‐constrained settings [35, 36].

Conversely, subjective norms and perceived behavioral control showed modest, nonsignificant gains (*p* = 0.0701; *p* = 0.4159), a pattern that departs from the TPB’s expectation that these constructs drive behavior change [37]. Coupled with small 1‐month declines in perceived support from supervisors (4.45–4.31) and colleagues (4.45–4.36), the results suggest training effects were not reinforced by the clinical environment pointing to hierarchical culture, workload pressures, and policy–practice gaps that blunt normalization of IPV screening [38–40]. This aligns with prior IPV training literature, indicating that without explicit leadership signals, clear procedures, protected time, and mentorship, post‐training motivation does not translate into durable practice [38, 39]. Together, these findings highlight the need for structured post‐training implementation supports to shift norms and perceived control and identify a priority for future longitudinal work on sustaining IPV screening beyond the initial intervention [39, 40].

The most compelling result was the sustained increase in actual IPV screening behavior, which rose from 22.7% pretraining to 72.7% at 1‐month follow‐up (*χ* ^2^ = 11.00, *p* < 0.001). Importantly, no significant decrease was observed between post‐training and follow‐up (*χ* ^2^ = 0.33, *p* = 0.564), demonstrating stable behavioral change. This sustained behavioral adoption and retention, fostered by the WHO curriculum’s integration of theoretical (12 h) and practical (18 h) components, builds upon previous studies that primarily showed only short‐term improvements [41]. Logistic regression further revealed that prior screening experience significantly predicted screening behaviors, with providers having previous experience 8.1 times more likely to screen for IPV (*p* = 0.014). This finding suggests experiential familiarity may be a more potent driver of behavioral adoption than attitudes or perceived norms alone, providing valuable insights for prioritizing practical experience in future training.

Culturally adapted, the training’s acceptability was strengthened by the Thai‐language adaptation and the program’s alignment with WHO’s emphasis on compassionate and empathic communication, critical to overcoming stigma and patriarchal norms in Thailand [42]. However, minor declines in some attitudes (e.g., perceived effectiveness of screening) 1 month post‐training indicate potential waning enthusiasm without reinforcement highlighting the need for ongoing institutional support to maintain positive behavioral changes.

### 4.1. Limitations

Several limitations must be considered in interpreting this study. First, the small sample size (*N* = 22) and lack of a control group limit the generalizability of findings and prevent definitive causal conclusions, typical constraints of quasiexperimental pilot studies. Potential selection bias is another concern, as participants may have been individuals already interested or motivated regarding IPV issues, further limiting generalizability. Additionally, the short follow‐up period of 1 month restricts conclusions regarding the long‐term sustainability of behavioral changes. Self‐reported data may also introduce social desirability bias, particularly given the sensitive nature of IPV in the Thai cultural context.

Furthermore, the theoretical component of the training was delivered online via Zoom, presenting challenges in maintaining participants’ sustained engagement, especially during the extended 12‐h theoretical component. Consequently, it remains uncertain whether engagement was fully maintained in this virtual environment. Finally, the training focused primarily on IPV screening skills, thus limiting its generalizability to broader aspects of IPV survivor care, such as ongoing counseling, safety planning, and coordination with community resources beyond initial screening.

### 4.2. Clinical Implementation Recommendations

Findings from this study highlight the potential for scaling up culturally tailored IPV screening training programs within Thailand’s healthcare system, especially in women’s health settings. To move from training to routine care, we specify operational steps. Embed discrete, privacy‐sensitive prompts in antenatal/gynecology/ED EHR templates with restricted‐access notes, a brief IPV screening field, and an auto‐generated referral order set (social work consult, safety planning, QR‐coded resource directory). Standardize a triage privacy workflow to secure 3–5 min alone with patients; when privacy is not possible, use a universal education script and defer direct inquiry. For sustainability, including IPV screening in orientation and annual competencies are needed; assign ownership to keep the referral directory current; and schedule brief mentorship check‐ins post‐rollout. Align institutional policies to provide protected time, clear documentation guidance (including sensitive‐note handling), and risk‐management procedures.

Future research should employ larger randomized samples and multicenter studies with extended longitudinal follow‐up periods (e.g., 6–12 months). Additionally, mixed‐methods approaches combining quantitative outcome measures and qualitative evaluations could provide deeper insights into barriers and facilitators to implementation, strengthening evidence to support nationwide adoption.

## 5. Conclusion

This pilot study demonstrates significant promise for culturally adapted IPV screening training in Thailand, highlighting the critical role of tailored education in enhancing healthcare providers’ engagement with IPV prevention. While significant increases in screening behavior indicate the intervention’s effectiveness, sustainability depends on supportive organizational environments and clear policy frameworks. As the first adaptation of the WHO IPV curriculum in Thailand, this research lays a robust foundation for scaling IPV interventions nationally. Future studies should utilize rigorous designs, larger randomized samples, extended longitudinal assessments, and mixed‐methods approaches to strengthen evidence supporting nationwide implementation, ultimately contributing to national health priorities and global efforts to eliminate violence against women.

## Ethics Statement

Ethical approval was granted by the Institutional Review Board (IRB) of Boromarajonani College of Nursing, Khon Kaen, Thailand (IRB‐BCNKK‐56‐2021).

## Consent

Participants were verbally consented using a standardized script and invited to ask questions about the research project. All participants consented to being audio‐recorded.

## Disclosure

All authors reviewed the results and approved the final version of the manuscript.

## Conflicts of Interest

The authors declare no conflicts of interest.

## Author Contributions

Research project conception and design: Tipparat Udmuangpia, Supawadee Thaewpia, Tina Bloom, Benjaporn Thitiyanviroj, Aimon Butudom, and Wannaporn Kampila; data collection: Tipparat Udmuangpia, Supawadee Thaewpia, Benjaporn Thitiyanviroj, and Wannaporn Kampila; draft manuscript preparation: Tipparat Udmuangpia, Supawadee Thaewpia, Tina Bloom, Benjaporn Thitiyanviroj, Aimon Butudom, Wannaporn Kampila, and Chiraporn Worawong.

## Funding

This research was funded by the Center for Addiction Studies, Thailand.

## Data Availability

The data that support the findings of this study are available upon request from the corresponding author. The data are not publicly available due to privacy or ethical restrictions.
